# Immune responses of *Helicoverpa armigera *to different kinds of pathogens

**DOI:** 10.1186/1471-2172-11-9

**Published:** 2010-03-03

**Authors:** Qian Wang, Yang Liu, Hong-Juan He, Xiao-Fan Zhao, Jin-Xing Wang

**Affiliations:** 1 School of Life Sciences, Shandong University; Jinan, Shandong 250100, China

## Abstract

**Background:**

Insects react against pathogens through innate immunity. The cotton bollworm *Helicoverpa armigera *(*H. armigera*) is an important defoliator and an extremely destructive pest insect of many crops. The elucidation of the mechanism of the immune response of *H. armigera *to various pathogens can provide a theoretical basis for new approaches to biologically control this pest.

**Results:**

Four kinds of pathogens *Bacillus thuringiensis*, *Klebsiella pneumoniae*, *Candida albicans*, and *Autographa californica *multiple nucleocapsid nucleopolyhedrovirus harbored green fluorescence protein and polyhedron (*Ac*MNPV-GFP) were used to challenge the insect. The cellular and humoral immune responses to the pathogens were analyzed in the challenged *H. armigera*. The results show that in the five kinds of haemocytes, only granulocytes phagocytized the Gram-negative and Gram-positive bacteria and fungi. All haemocytes can be infected by *Ac*MNPV. Fourteen immune-related genes including pattern recognition receptors (PRRs) such as peptidoglycan recognition proteins (*HaPGRP *and *HaPGRP C*) and Gram-Negative Bacteria-Binding Protein (*HaGNBP*), and antimicrobial peptides (AMPs) such as *cecropin-1, 2 *and *3 *(*HaCec-1, 2 *and *3*), *lysozyme *(*HaLys*), attacin (*HaAtt*), *gallerimycin-like *(*HaGall*), *gloverin-like *(*HaGlo*), *moricin-like *(*HaMor*), cobatoxin-like (*HaCob*), *galiomicin-like *(*HaGali*), and *immune inducible protein *(*HaIip*) appeared in different expression profiles to different pathogen infections. The transcripts of 13 immune related genes (except *HaPGRPC*) are obviously up-regulated by Gram-positive bacteria. *HaCec-1 and 3, HaMor, HaAtt, HaLys*, *HaIip*, *HaPGRP *and *HaGNBP *are greatly up-regulated after fungal infection. *HaGNBP, HaCec-2, HaGall, HaGlo, HaMor, HaCob, HaGali *obviously increased in Gram-negative bacterial infection. Only five genes, *HaGNBP, HaCec-1*, *HaGali*, *HaGlo*, and *HaLys*, are weakly up-regulated after viral infection. The AMP transcripts had higher expression levels than the PRR transcripts after the microbial challenge.

**Conclusions:**

These data suggest that the granulocytes are the major phagocytes in *H. armigera*. All haemocytes can be infected by *Ac*MNPV. The transcripts of 14 immune related genes have different expression patterns in *H. armigera *infected by different pathogens, which means that the immune-related genes may have different functions against various kinds of pathogens.

## Background

The immune system is generally divided into innate and adaptive immunity. Innate immunity is characterized by quick reactions that cause immediate immune responses. In contrast, adaptive immunity is a slow reaction with high specificity and memory. Adaptive immunity has remarkable specificity based on somatic gene rearrangement and hypermutation, leading to an extremely large repertoire of T- and B-cell receptors and antibodies. Such adaptive immunity is restricted to jawed vertebrates. Invertebrates only rely on their innate immune defenses.

The innate immune system of insects relies on both humoral and cellular responses [1]. Haemocytes are the primary mediators of cell-mediated immunity in insects including phagocytosis, nodulation, encapsulation, and melanization. The humoral response of innate immunity includes three steps: 1) identification of pathogen-associated molecular patterns (PAMPs) on pathogens by pattern recognition receptors (PRRs) [2]; 2) activation of the regulatory pathways; and 3) production of immune effectors including cellular phagocytosis and molecular effectors such as antimicrobial peptides (AMPs) [3].

Innate immune recognition is an active area of research that has quickly developed over the last few years along with the study of Toll receptors [4-6]. Unlike adaptive immunity, which recognizes every antigen, innate immunity recognizes the conserved Pathogen-associated molecular patterns (PAMPs) of the pathogens by the PRRs of the hosts [7]. Several PRRs have been reported such as peptidoglycan recognition proteins (PGRPs), thioester-containing proteins (TEPs), Gram-negative binding proteins (GNBPs), multidomain scavenger receptors (SCRs), C-type lectins (CTL), galectins (GALE) [5], and Down syndrome cell adhesion molecule (DSCAM) [8].

Effector molecules such as AMPs are found in most of plants and animals, and their functions in innate immunity are well investigated [9,10]. Approximately 1,000 varieties of AMPs have been reported from vertebrates, invertebrates, human beings, and plants since the Boman group reported the first cecropin from *Hyalophora cecropia *[11]. In most insects, AMPs are synthesized in the fat body and haemocytes and are then released to the haemolymph.

Insect innate immunity has been studied in *D. melanogaster*, *Anopheles gambia*, and *Maduca sexta *[12,13], but fewer studies have been conducted on the cotton bollworm, *Helicoverpa armigera. H*. *armigera *is a worldwide pest that has developed strong resistance to chemical and biological pesticides. New approaches to control this pest can be designed if the mechanism of its immune responses to pathogens is understood. We have reported the function of a pattern recognition receptor (C-type lectin) in the insect [14,15]. To further investigate the mechanism of *H. armigera's *response to pathogen infections, its cellular responses to four kinds of pathogens, namely, Gram-positive bacterial, Gram-negative bacterial, fungal, and viral pathogens which are *Bacillus thuringiensis *(G^+^-bacterium), *Klebsiella pneumoniae *(G^-^-bacterium), *Candida albicans *(fungus), and *Autographa californica *multiple nucleocapsid nucleopolyhedrovirus harbored green fluorescence protein and polyhedron (*Ac*MNPV-GFP) (virus), respectively, were investigated. Simultaneously, quantitative real-time PCR (qRT-PCR) analysis was performed at different times after the challenge by these pathogens. Fourteen immunity-related genes including PRRs and antimicrobial peptide genes were chosen for the analysis. The results demonstrated that the innate immune responses of *H. armigera *larvae to the pathogens included cellular phagocytosis and up-regulated expression of immune effector genes.

## Results

### Cellular responses to pathogens in vivo

**Figure 1:**
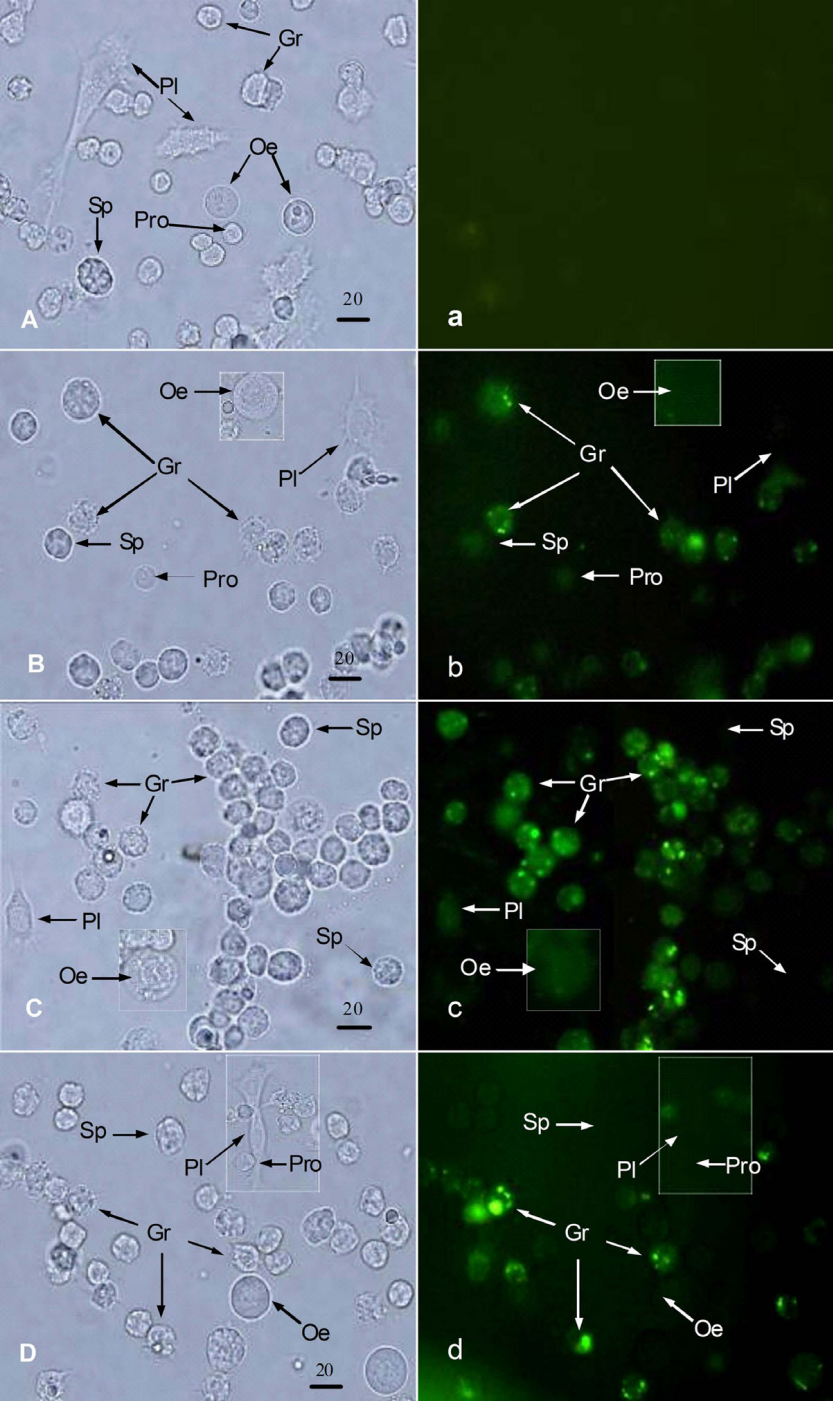
**Phagocytosis of the pathogens by the haemocytes**. Pathogens were injected into the haemocoel (1× 10^5 ^cfu/larva) of *H. armigera *for 3 h and then haemocytes were collected and examined under the microscope. A, a, Haemocytes from normal larvae; B, b, Haemocytes from the larvae of *B. thuringiensis *injection, C, c, Haemocytes from the larvae of *K. pneumoniae *injection, D, d, Haemocytes from the larvae of *C. albicans *injection. The capital characters A, B, C, and D are the pictures under white light. The small characters a, b, c, and d are the pictures under fluorescent light. The rule is 20 μm. Pl, plasmatocytes; Gr, granulocytes; Oe, oenocytoids; Sp, spherulocytes.

Five kinds of cells were distinguished in *H. armigera*, namely, prohaemocytes (Pr), plasmatocytes (Pl), granulocytes (Gr), oenocytoids (Oe), and spherulocytes (Sp) (Fig. 1A). After the injection of acridine orange labeled bacteria and fungi, it was observed that only the granulocytes could phagocytize the pathogens including G^+ ^organism *B. thuringiensis*, the G^- ^organism *K. pneumoniae*, and the fungus *C. albicans*. Other haemocytes did not phagocytize (Fig. 1B-d). This result indicated that the granulocytes were the major phagocytes in *H. armigera*.

**Figure 2:**
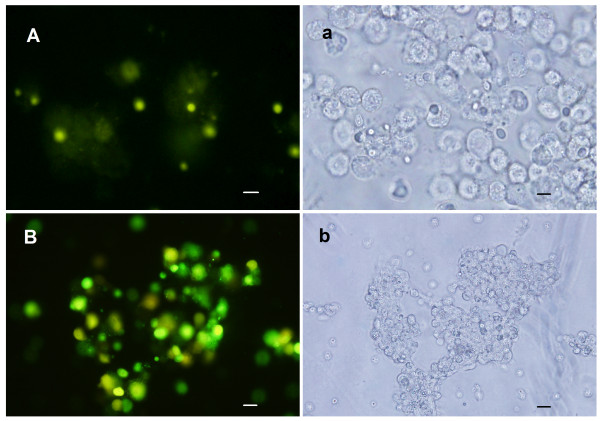
**Infection of haemocytes by *Ac*MNPV-GFP in vivo**. A, a, Haemocytes from larvae 36 h post infection; B, b, Haemocytes from larvae 6 day post infection. Rules are 20 μm, respectively. The capital characters A, B, C, and D are the pictures under fluorescent light. The small characters a, b, c, and d are the pictures under white light.

When the budded virus, *Ac*MNPV-GFP was injected into the larval haemocoel, haemocytes released green fluorescence at 36 h after injection. The morphology of the haemocytes was changed and could not be identified as a recognizable cell type. The green fluorescence increased in intensity proportional to infection time till 6 d post infection. The haemocytes appeared aggregated and destroyed. However, the larvae did not die, but were arrested in the metamorphically committed stage till 6th instar 6 d. These results suggested that the haemocytes could be infected by the *Ac*MNPV-GFP virus and the virus could replicate inside the insect body (Fig. 2).

### Immune related genes obtained by random sequencing

**Figure 3:**
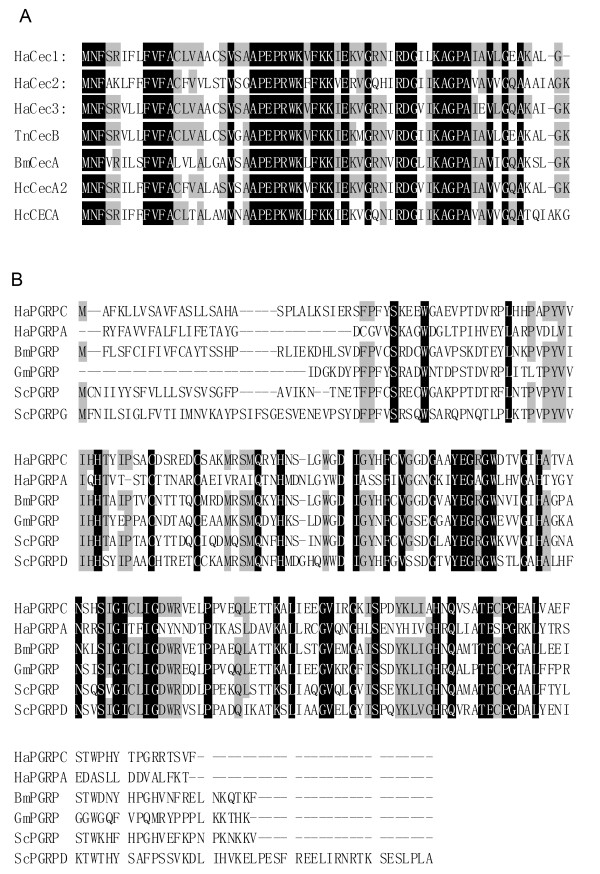
**Sequence alignment of cecropins and PGRPs from *H. armigera *and other species**. (A) Alignment of complete sequence of cecropin-1, 2, and 3 from *H. armigera *and cecropin sequences from other species. TnCec B: *Trichoplusia ni *(GenBank No. ABV68872); BmCecA: *Bombyx mori*, (GenBank No. NP_001037462); HcCecA2: *Hyphantria cunea*, (GenBank No. P50722); HcCecA: *Hyalophora cecropia*, (GenBank No. P01507). (B) Sequence alignment of PGRPs from *H. armigera *and other species. BmGRBP: *Bombyx mori *(GenBank No. NP_001036858); GmPGRP: *Galleria mellonella *(GenBank No. CAL36191); ScGRBP: *Samia cynthia ricini *(GenBank No. BAF03521); ScGRBPD: *Samia cynthia ricini *(GenBank No. BAF74637). The identical residues were in white of black background.

Four thousand clones from the cDNA library were sequenced. More than 20 expressed sequence tags (ESTs) of immune related genes were obtained, including pattern recognition receptors: *Peptidoglycan recognition proteins *(*HaPGRPA*, GenBank accession No.: GU182905; and *HaPGRPC*, GU182906), *Gram-negative bacteria binding protein *(*HaGNBP*, GU182914) and C-type lectins (*HaLec*, DQ533877); antimicrobial peptides: *cecropin-*1 (GU182916), 2 (GU182909), and 3 (GU182910)(*HaCec-*1,2 and 3),*attacin (HaAtt*, GU182917), *lysozyme (HaLys*, GU182915),*gallerimycin-like (HaGall*, GU182913),*gloverin-like (HaGlo*, GU182908),*moricin-like (HaMor*, GU182911),*cobatoxin-like (HaCob*, GU182912),*galiomicin-like (HaGali*, GU182907)*AMPs *and *immune inducible protein (HaIip*, DQ875243). The sequences of 3 cecropins (HaCecs) and 2 PGRPs (HaPGRPs) were showed in Fig. 3.

### Molecular responses of *H. armigera *to the pathogens

To analyze the molecular responses of *H. armigera *to 4 different pathogens, quantitative real-time PCR was performed. The time course expression patterns of 14 genes were examined including three upstream PRR genes such as *HaPGRP and HaPGRP C*, and *HaGNBP*, and 11 downstream effector genes such as *HaCec*-*1,2 *and *3*, *HaLys*, *HaAtt, HaGall, HaGlo, HaMor, HaCob, HaGali *and *HaIip*.

**Figure 4:**
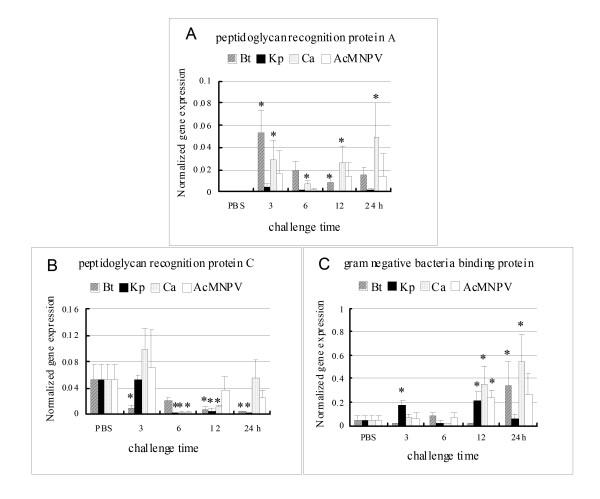
**The time course expression of PRR transcripts to infection of 4 pathogens examined by Real-time PCR**. A, B and C show the expression profiles of *HaPGRPA*, *HaPGRPC*, *HaGNBP *respectively. *Bt, B. thuringiensis*; *Kp, K. pneumoniae*; *Ca, C. albicans*; and *Ac*MNPV-GFP. (* means P < 0.05, indicating the significant differences between PBS and pathogens challenge at one point in time. PBS controls were performed at time points from 3 h to 24 h for every transcripts, and no significant variation was observed in the transcription level).

The expression patterns of the three pattern recognition protein genes had some differences responding to the pathogens. The expression of *HaPGRP *was increased 3 h post-injection of the G^+ ^bacteria and fungi; there was no significant increase in expression after G^- ^bacteria injection and virus injection compared with the PBS-injected insects (Fig. 4A). In contrast, *HaPGRP C *showed a trend of decreased expression after the injection of G^+ ^bacteria, G^- ^bacteria, fungi, or virus (Fig. 4B). The *HaGNBP *was up-regulated to the highest expression sequentially by four pathogens 3 h post-G^- ^bacterial injection, 12 h post- G^+ ^bacterial, fungal and -viral injection. The expression level of *HaGNBP *is higher than *HaPGRP *(Fig. 4C). These results indicated that the expressions of PRRs were regulated after the infections of the pathogens.

**Figure 5:**
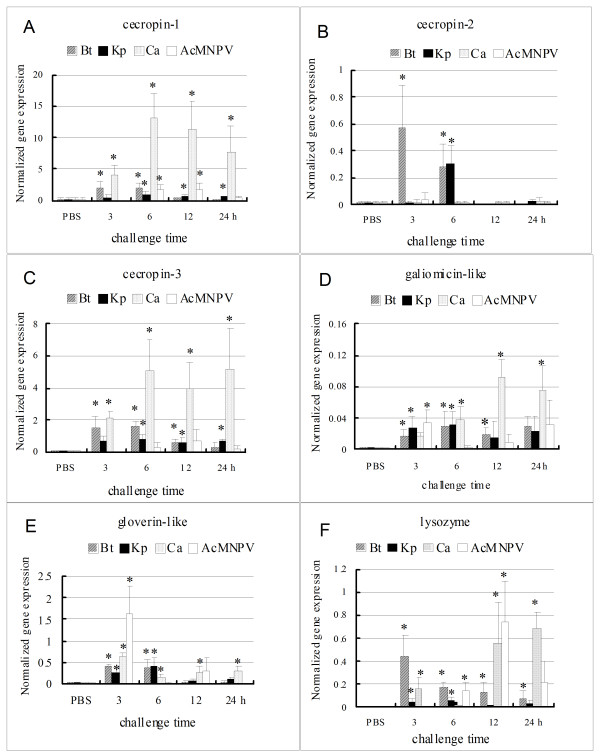
**The time course expression of 6 AMP transcripts to different pathogen infection examined by Real-time PCR**. One panel shows the transcript levels of an immune related gene to 4 kinds of pathogens. The symbols and abbreviations are the same as in Fig. 4. A shows the expression pattern of *cecropin-1(HaCec-1)*; B: *cecropin-2(HaCec-2)*; C: cecropin-3 *(HaCec-3)*; D: *Galiomicin-like antimicrobial peptide(HaGali)*; E: *gloverin-like (HaGlo)*; F: *lysozyme (HaLys)*.

Three *cecropins (HaCec-1, 2 *and *3) *had different expression patterns responding to the pathogens. *HaCec-1 *and *3 *were up-regulated by four or three pathogens, respectively. However, higher expression levels of *Ha*C*ec-1 *and *3 *were found in the fungal injected larvae (Fig. 5A, C). In contrast, *HaCec-2 *was only up-regulated by two kinds of bacteria with lower expression levels, 3 h post-injection by G^+ ^bacteria and 6 h post-injection by G^- ^bacteria. Almost no variation was observed for fungal and viral challenge compared with the mock (PBS) challenge (Fig. 5B). The *HaCec-1 *and *3 *had high similarities in amino acid sequence comparison (Fig. 3A), therefore they had similar expression pattern after pathogen infection. These results also suggested that *Ha*C*ec-1 *and *3 *responded to fungus well and that *HaCec-2 *responded to bacteria well.

The *HaGali*, *HaGlo*, and *HaLys *were up-regulated by four microbial challenges. However, they exhibited different expression patterns. The expression of *HaGali *was up-regulated by four microbial challenge 3 h post-injection, with the highest expression after fungal challenge at 12 h (Fig. 5D). The mRNA of *HaGlo *was also up-regulated by four microbes 3 h post-injection but with the highest expression after viral challenge at 3 h (Fig. 5E). The expression of *HaLys *was up-regulated by four pathogens with different patterns. It was up-regulated 3 h post-injection of the G^+ ^bacteria and gradually recovered to normal level from 6 h to 24 h, and it was up-regulated 3 h post-injection of the fungus and gradually increased to a higher level from 12 h to 24 h post-injection. Similar to fungus induction, *HaLys *was up-regulated by *Ac*MNPV 6 h post-injection and reached the highest level at 12 h post-injection. *HaLys *showed higher responses to virus and fungus than to bacteria (Fig. 5F). The evidence suggested that the genes had different responses to different pathogens. *HaGali *responded well to fungus;*HaGlo *responded well to virus; and *HaLys *responded well to both fungus and virus.

**Figure 6:**
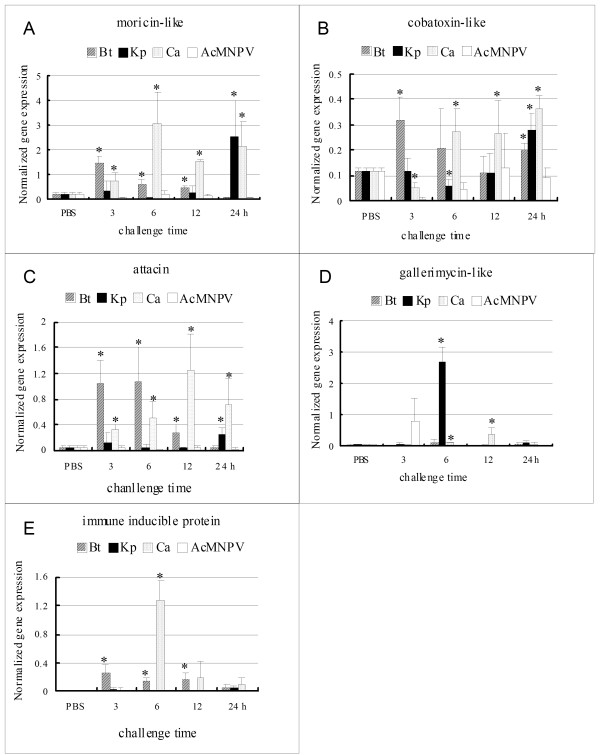
**The time course expression of 5 AMP transcripts to different pathogen infection examined by Real-time PCR**. One panel shows the transcript levels of an immune related gene to 4 kinds of pathogens. The symbols and abbreviations are the same as in Fig. 4. A: *moricin-like (HaMor)*; B: *cobatoxin-like (HaCob)*; C: *attacin (HaAtt)*: D: *gallerimycin-like (HaGall)*; E: *immune inducible protein (HaIip)*.

The *HaMor*, *HaCob *and *HaAtt *were up-regulated by bacteria and fungus but not by virus. The expression of *HaMor *was up-regulated to the highest level by G^+ ^bacteria 3 h post-injection, by fungus 6 h post-injection, and by G^- ^bacteria 24 h post-injection (Fig. 6A). The expression of *HaCob *was up-regulated to the highest levels 3 h post-G^+ ^bacterial injection, 6 h post-fungal injection, and 24 h post-G^- ^bacterial injection (Fig. 6B). *HaAtt *was increased 3 h to 6 h post-injection of the G^+ ^bacteria and gradually decreased to normal level from 12 h to 24 h post-injection. In contrast, *HaAtt *appeared to have an increase trend from 3 h to 12 h post-injection of fungus. There was a low-level increase of the *HaAtt *at 24 h post-G^- ^bacterial injection. The expression levels of *HaAtt *to fungus and G^+ ^bacteria were much higher than to G^- ^bacteria (Fig. 6C). These results suggested that the genes had different initiation response times to different pathogens and had different expression patterns after infection of various pathogens.

The *HaGall *and *HaIip *only responded to two pathogens. *HaGall *was greatly up-regulated by G^- ^bacteria 6 h after injection and was weakly up-regulated by fungus at 12 h post-injection (Fig. 6D). *HaIip *was greatly up-regulated by fungus 6 h after injection and was weakly up-regulated by G^+ ^bacteria at 3 h to 12 h post-injection (Fig. 6E). These two genes could play roles against G^- ^bacteria infection and fungus infection, respectively.

## Discussion

This work first investigated the immune responses at the cellular and humoral levels of *H. armigera *to four pathogens, *B. thuringiensis*, *K. pneumoniae*, *C. albicans*, and *Ac*MNPV-GFP, which represent G^+ ^bacterial, G^- ^bacterial, fungal, and viral pathogens, respectively. The results revealed that the innate immunity of *H. armigera *larvae responded both on cellular and humoral levels against these pathogens.

Regarding the functions of haemocytes relating to innate immunity, it is known that plasmatocytes form the bulk of capsules around foreign bodies. Granular haemocytes phagocytize foreign pathogens, and oenocytoids produce phenoloxidases (PO), which are responsible for haemolymph melanization [16]. Plasmatocytes are involved in the phagocytosis of non-self microsphere beads, whereas granulocytes are involved in the phagocytosis of self-dead cells. Other studies showed that phagocytes of *Manduca sexta *had similar functions as vertebrate phagocytes [17]. This study demonstrated that granulocytes were the major cells that phagocytized G^+ ^and G^- ^bacteria, and fungi. No phagocytosis function was observed for other kinds of haemocytes in *H. armigera*. This suggested that haemocytes played different roles in different insect species.

In addition to cellular immunity, humoral immunity also plays significant role in insect immunity, which involves the induction of a robust AMPs that kill invading pathogens [9]. Eleven AMP genes were found from *H. armigera *in this study: *HaCec-1*, *2 *and *3*, *HaGall, HaGlo, HaLys, HaMor, HaCob, HaAtt, HaGali*, and *HaIip*. They belong to 9 kinds of AMPs.

Some functions of these AMPs are known in other species. For example, the cecropins are linear, cationic AMPs that cause lysis of both Gram-positive and Gram-negative bacteria [18]. *HaCec-1 *appeared to have very high expression levels in fungus *C. albicans*-injected larvae and *Ac*MNPV-injected larvae in addition to G^+ ^and G^- ^bacteria, suggesting that *HaCec-1 *not only responded to G^+ ^and G^- ^bacteria but also to fungus and virus, and might play very important roles in *H. armigera *immunity.

Furthermore, Galiomicin, a defensin first found in *Galleria mellonella*, has antibacterial and antifungal activities [19]. The results of this study were consistent to the report except for the detection of some up-regulation of *HaGali *after virus infection. Gloverins, a family of glycine-rich polypeptides, are effective against G^- ^bacteria and are inactive against G^+ ^bacteria, yeasts, mammalian cell lines, and pestivirus [20]. However, up-regulation of this gene was detected after four kinds of pathogens were injected into *H. armigera *including G^+ ^and G^- ^bacteria, fungus, and virus. The highest expression of *HaGlo *after *Ac*MNPV virus injection suggests that this gene may play a role in antivirus defense. Lysozymes are a type of hydrolase which can digest the peptidoglycan layer of the bacteria cell wall by hydrolyzing the β-1,4-glycosidic linkage between N-acetylglucosamine and N-acetylmuramic acid. Lysozymes also hydrolyze the β-1, 4-linkages of chitooligosaccharides in the fungal cell wall. Therefore, lysozymes have antibacterial and antifungal activities [21]. In addition to the antibacterial and antifungal activities, the results of this study indicate that *HaLys *also highly respond to virus, which implies its role in antivirus defense.

Moricin is a highly basic AMP first reported in the silkworm *Bombyx mori*. It shows antibacterial activity against several G^- ^and G^+ ^bacteria [22]. The results in *H. armigera *showed that *HaMor *also had a higher expression after *C. albicans *(fungus) induction, suggesting that *HaMor *played a role in antifungus response. Cobatoxin is a toxin present in the venom of the *Centruroides noxius *scorpion that blocks two K^+^-channel subtypes: voltage-gated and Ca^2+^-activated channels [23]. The results of this study show *HaCob *responded to G^- ^and G^+ ^bacteria and fungus infections in *H. amigera*. Attacin is an AMP, originally isolated from the immune haemolymph of *Hyalophora cecropia*. The antibacterial effect of attacin was found to be limited to some species of Gram-negative bacteria [24]. In *H. armigera*, *HaAtt *responded to G^+ ^bacteria and fungus better than G^-^bacteria, suggesting that *HaAtt *participated in the anti-G^+ ^bacteria and fungus response in addition to G^- ^bacteria.

Gallerimycin is an antifungal peptide from the greater wax moth *G. mellonella*. Its expression is up-regulated after stimulation with bacterial lipopolyscaccharides (G^- ^bacteria). Gallerimycin is active against the entomopathogenic fungus *Metarhizium anisopliae *but not against yeast and Gram-negative or Gram-positive bacteria in *G. mellonella *[25]. *Helicoverpa *gallerimycin-like AMP had a very high expression after the G^- ^bacteria (*K. pneumoniae*) challenge, suggesting that it played a role in G^- ^bacteria defense. *HaIip *is an antimicrobial peptide with Knot1 domain representing plant lectins http://smart.embl-heidelberg.de. The up-regulation of *HaIip *in *H. armigera *after the induction of fungus (*C. albicans*) suggests that it played an important role in antifungal defending.

Innate immunity was formerly thought to be a non-specific immune response characterized by phagocytosis. However, innate immunity has considerable specificity and is capable of discriminating between self and non-self, as proposed in the concept of PRRs of the host. These PRRs can recognize conserved PAMPs shared by large groups of microorganisms, therefore successfully defending invertebrates and vertebrates against infection [2]. PGRP was first purified from silkworm (*Bombyx mori*) hemolymph based on the high affinity of PGRP to bacterial peptidoglycans[26]. GNBPs have been originally described in the silkworm *B. mori*, and one of them is known to bind strongly to the surface of Gram-negative bacteria[27]. The expression of *HaPGRP *was up-regulated by G^+ ^bacteria and fungi; the *HaGNBP *was up-regulated by four kinds of pathogens. These results indicated that the PRRs in *H. armigera *might have different functions in the pattern recognition.

The results of expression patterns revealed that most examined genes were significantly up-regulated after infection by pathogens. However, the responses of the genes to different kinds of pathogens showed specific characters. Although some genes could respond to all four tested pathogens, the intensity of their expression to different pathogens varied. For instance, *HaCec-1*, *HaCec-3*, and *HaIip *were greatly up-regulated after fungi infection, which suggests that these genes mostly responded to fungal infection in *H. armigera*, although they also did respond to other pathogens. *HaCec-2 *was up-regulated by G^+ ^and G^- ^bacteria, which suggests that it responds to bacteria. *HaGall *obviously increased in G^- ^bacterium (*K. pneumoniae*) infection, and *HaGlo *also obviously increased in *Ac*MNPV infection, which might imply that these genes were involved in response to the Gram-negative bacterium or virus, respectively. In addition, only five genes were up-regulated after virus infection: *HaGNBP, HaCe1*, *HaGali*, *HaGlo*, and *HaLys*, but the expression levels of the genes against the virus were much lower except *HaGlo *and *HaLys*. These phenomena suggest that the same AMP gene might respond to different pathogens by different expression patterns.

Lemaitre et al. [28] found that the challenge of fruit flies with a fungus results in the biosynthesis of anti-fungal AMPs, whereas the challenge by a G^- ^bacterium results in the up-regulation of AMPs appropriate for the destruction of such bacteria. It seems that there is no strict correspondence in the immune response of *H. armigera *against different pathogens in mRNA level, although there are specific characters as described above. The expression patterns and functions of the immune related genes at protein levels need to be investigated in the future work.

There is growing interest in antiviral mechanism in *Drosophila *[29-31]. Dostert *et al*. identified 140 genes that were upregulated in adult *Drosophila *24 and 48 h post injection with *Drosophila *C virus. Two thirds of these genes were not upregulated in response to bacterial or fungal infections. However, most genes encoding antimicrobial peptides regulated by the Toll and Imd pathways were not upregulated or were only weakly upregulated by DCV infection [31]. Same situation was found in *H. armigera*, only *HaGlo *and *HaLys *were weakly upregulated by viral challenge. This might suggest that the insect used a distinct mechanism to combat viral infection. *H. armigera *is a good model to analyze the viral infection. It is worth further investigation for the antiviral mechanism.

## Conclusions

Five kinds of hemocytes were distinguished in *H. armigera*, the granulocytes in the insect are the major phagocytes. All haemocytes can be infected by *Ac*MNPV. The expression patterns of 3 upstream PRR genes and 11 downstream effector genes in the insect were analyzed by qRT-PCR. The transcripts of 14 immune related genes have different expression patterns in *H. armigera *infected by different pathogens. Most of the genes were upregulated by bacteria and fungi, and only five genes were upregulated by the virus *Ac*MNPV. These data suggested that the immune-related genes might have different functions against various kinds of pathogens.

## Methods

### Chemicals

The chemicals were obtained from the following separate companies: Unizol reagent (Biostar Company, Shanghai, China); Reverse Transcriptase (RT) Kit (Promega Biosciences, Madison WI, USA); PCR purification kit (Shengong, Shanghai, China); Ex Taq Polymerase and SYBR^®^Premix EX Taq (TaKaRa Biotech, Dalian, China); and 4'-6-Diamidino-2-phenylindole dihydrochloride (DAPI, 1 μg/ml in water, San Jose, United States). Grace's medium was from Invitrogen (United States).

### Insect

The eggs of *H. armigera *were obtained from the Wuhan Institute of Virology, Chinese Academy of Science, Wuhan, China. After incubation, the larvae were maintained in the laboratory at 26 ± 1°C under light and dark conditions for 14:10 h and were reared on the artificial diet described by Zhao et al. [32].

### Microbial pathogens

*Bacillus thuringiensis*, *Klebsiella pneumoniae*, and *Candida albicans *were from Shandong Agricultural University. *Ac*MNPV-GFP was constructed in the researchers' laboratory [33].

The G^+ ^bacterium *B. thuringiensis*, G^- ^bacterium *K. pneumoniae*, and fungus *C. albicans *were cultured overnight in 5 ml Luria-Bertani medium (LB, 1% trytone, 0.5% yeast extract, 1% NaCl in w/v, pH 7.0). The virus *Ac*MNPV-GFP was cultured in Sf21cells with Grace's medium.

### Phagocytosis of the pathogens in vivo in *H. armigera* larvae

*B. thuringiensis*, *K. pneumoniae*, and *C. albicans *were collected from the LB medium by centrifugation at 6,000 rpm for 10 min, fixed in 95% ethanol for 10 min, suspended in PBS (140 mM NaCl, 2.7 mM KCl, 10 mM Na_2_HPO_4_, 1.8 mM KH_2_PO_4_, pH7.4), added to acridine orange at 1 μg/ml for 10 min, centrifuged, and washed in PBS to clear. The microbes stained with acridine orange were injected into the haemocoels of three 5th instar 24 h larvae. Each larva was injected with 1 × 10^5 ^colony forming units (cfu) in 5 μl of PBS. The haemocytes were collected by cutting the proleg of the caterpillar 3 h post-injection, mixed a 200 μl PBS with 1 mM reduced glutathione and covered by glass cover slip, and observed under microscope.

### Infection of larvae by the pathogens and extraction of RNA

*B. thuringiensis*, *K. pneumoniae*, and *C. albicans *were collected from the LB medium by centrifugation at 6,000 rpm for 10 min, washed three times with PBS, and then resuspended in PBS. The number of pathogens was calculated by a haemocytometer. The pathogens were injected into the haemocoels of six 5th instar 24 h larvae. Each larva was injected with 1 × 10^5 ^colony forming units (cfu) in 5 μl of PBS. The budded virus (BV) of *Ac*MNPV-GFP, amplified in Sf21 cells, was injected into the haemocoels at a concentration of 1 × 10^7^pfu/5 μl/larva. AcMNPV virus was quantitated using plaque forming units following the viral plaque assay in the instruction manual of Bac-to-Bac Baculovirus Expression Systems of Invitrogen http://wolfson.huji.ac.il/expression/bac.pdf. Briefly, the monolayers of Sf9 cells were prepared in 6-well plates, Eight-log serial diluted virus (10^-1^- 10^-8^) were produced and the diluted virus (10^-3^- 10^-8^) were added to each well of the plates respectively and incubate 1 h at room temperature. Sequentially the virus inoculum was removed from the wells and replaced with 2 ml diluted agarose and incubated at 27°C in a incubator for 5 days. The number of plaques was counted. The titer of the virus can be calculated by the following formula: pfu/ml of the virus = 1/dilution factor × number of plaques × 1/(ml of inoculum/plate). Controls were injected with PBS or Grace's medium, respectively, for the bacteria and virus. A fluorescence microscope (Olympus BX51) was used to observe the green fluorescence. The total RNAs were extracted from the whole bodies of three randomly chosen larvae per treatment to normalize the individual difference and then they were reverse transcribed to complementary deoxyribonucleic acid (cDNA) for quantitative real-time PCR analysis.

### cDNA library construction

The total RNA was extracted from whole body of *H. armigera *using Unizol reagent following the manufacture's instructions (Biostar, Shanghai, China). Messenger RNA (mRNA) was extracted with the PolyATract mRNA isolation system (Promega, USA). The mRNAs were used to construct a cDNA library. The Creator SMART cDNA Library Construction Kit (Clontech, USA) was used for the cDNA library construction following the manufacturer's instructions. The double strand cDNA was digested and ligated with the pDNR-LIB vector, and then transformed into competent DH5α cells. Individual colonies were randomly selected, and plasmid was extracted for sequencing from the 5'-ends (Beijing Genomics Institute, China).

### Quantitative real-time PCR (qRT-PCR)

The primers of the detected genes were designed from the specific sequences of the genes obtained by cDNA library sequencing in the laboratory (Table 1). The quantitative real-time PCR was performed following the manufacturer's instruction of the SYBR Premix Ex Taq kit (Takara, Japan) with a real-time thermal cycler (Bio-Rad, Hercules, CA). The cDNA templates, obtained in method 2.5, were first examined by semi-quantitative RT-PCR for the qualities of the cDNAs and the gene primers by β-actin and every examined gene. The cDNA templates were then diluted 1:20 in distilled water, and the primers (100 μM) were diluted 1:100 in distilled water. The reaction mixture SYBR^®^Premix EX mixture 5 μl, primer 2 μl each, template 1 μl, water 2 μl was mixed on ice. The tubes with the reaction mixtures were put into the real-time PCR machine, and the following procedure was followed: one cycle of 95°C for 3 min, 40 cycles of 95°C for 15 s, 62°C for 50 s, read, 72°C for 2 s, read, 82°C for 2 s, read, 72°C for 10 min. The qRT-PCR data from three repeats were analyzed using the Opticon Monitor 2. Furthermore, the expression level of the immune-related gene was analyzed using the comparative C_T _method. In this method, the discrepancy between the C_T _for the gene and β-actin (ΔCT) were calculated to normalize the variation in the amount of cDNA in each reaction. The data obtained from three repeats were calculated by 2^-ΔCt ^and statistically analyzed by student *t*-test. A significant difference was accepted at *P *< 0.05.

**Table 1:** Primers for Quantitative Real-time PCR

Name of gene	Name of primer	Primer sequence	cDNA length (bp)
*PGRP*	HaPGRPF HaPGRPR	5'-ccagatgtgctgagatcgtg-3' 5'-tttgccgttaccacctacaa-3'	101
*PGRP C*	HaPGRPcF HaPGRPcR	5'-agctgaggtacccacagacg-3' 5'-ccagcccaagctgttatgat-3'	151
*GNBP*	HaGNRPF HaGNRPR	5'-caccttgcattacggacctt-3' 5'-tgttacacgtcccagttcca-3'	166
*galiomicin*	HaGaliF HaGaliR	5'-tggtgaagagctaccgttcc-3' 5'-acgcagctaccaatcagctt-3'	109
*gloverin*	HaGloF HaGloR	5'-gcaagacatcttcaacgacca-3' 5'-tccttgtacacatcaagactgg-3'	150
*lysozyme*	HaLysF HaLysR	5'-gaaggactgcaatgttacttg-3' 5'-gcctcgaacttgtggcgtttg-3'	99
*Cecropin-1*	HaCecF HaCecR	5'-gtttggtagcagcgtgcag-3' 5'-gcttcaccgaggactgctat-3'	136
*cecropin-2*	HaCecAF HaCecAR	5'-tgtcttcgcttgttttgtgg-3' 5'-atcacgaatgtgctgaccaa-3'	103
*cecropin 3*	HaCecBF: HaCecBR	5'-gttgttcgtgttcgcgtgt-3' 5'-accgtccctgatgttacgac-3'	112
*moricin*	HaMorF HaMorR	5'-gcattactggtgccatctga-3' 5'-ctatgttgatcgcccggagt-3'	103
*cobatoxin*	HaCobF HaCobR	5'-tgtgctagttgttataagtgccatt-3' 5'-ctacctgcaccgagttgtca-3'	122
*attacin*	HaAttF HaAttR	5'-gagtgggagcttcattaggg-3' 5'-cgaggagcgttaaagtccag-3'	120
*gallerimycin*	Ha-GallF1 Ha-GallR1	5'-acaagggccacctcttccag-3' 5'-aagtgcagtatccgccagac-3'	92
*immune inducible protein*	HaIipF HaIipR	5'-cttatagggtgcgaccaacg-3' 5'-acgtccgagttacagcgaag-3'	161
*Ha-β-actin*	HaActinF HaActinR	5' -cctggtattgctgaccgtatgc-3' 5' -ctgttggaaggtggagagggaa-3'	150

## Authors' contributions

QW made contributions to acquisition, analysis and interpretation of all qRT-PCR data and manuscript draft writing. YL performed experiments of cellular responses of *H. armigera *to pathogen infections. HJH performed the cDNA library construction. XFZ and JXW made contributions to the study design, data interpretation and helping to draft the final version of the manuscript. All authors read and approved the final manuscript.

## References

[B1] MD Lavine Strand MR Insect hemocytes and their role in immunity Insect Biochemistry and Molecular Biology 2002 32 1295 1309 10.1016/S0965-1748(02)00092-9 12225920

[B2] Medzhitov R Janeway C Innate immune recognition: mechanisms and pathways Immunological Reviews 2000 173 89 97 10.1034/j.1600-065X.2000.917309.x 10719670

[B3] Schulenburg H Kurz CL Ewbank JJ Evolution of the innate immune system: the worm perspective Immunol Rev 2004 198 36 58 10.1111/j.0105-2896.2004.0125.x 15199953

[B4] Akira S TLR signaling Curr Top Microbiol Immunol 2006 311 1 16 full_text 17048703 10.1007/3-540-32636-7_1

[B5] Christophides GK Vlachou D Kafatos FC Comparative and functional genomics of the innate immune system in the malaria vector Anopheles gambiae Immunol Rev 2004 198 127 48 10.1111/j.0105-2896.2004.0127.x 15199960

[B6] Rast JP Smith LC Loza-Coll M Hibino T Litman GW Review - Genomic insights into the immune system of the sea urchin Science 2006 314 952 956 10.1126/science.1134301 17095692 PMC3707132

[B7] Janeway CJ The role of self-recognition in receptor repertoire development Immunol Res 1999 19 107 18 10.1007/BF02786480 10493166

[B8] Watson FL Puttmann-Holgado R Thomas F Lamar DL Hughes M Kondo M Rebel VI Schmucker D Extensive diversity of Ig-superfamily proteins in the immune system of insects Science 2005 309 1874 1878 10.1126/science.1116887 16109846

[B9] Bulet P Stocklin R Menin L Anti-microbial peptides: from invertebrates to vertebrates Immunological Reviews 2004 198 169 184 10.1111/j.0105-2896.2004.0124.x 15199962

[B10] Zasloff M Antimicrobial peptides of multicellular organisms Nature 2002 415 389 95 10.1038/415389a 11807545

[B11] Hultmark D Steiner H Rasmuson T Boman HG Insect immunity Purification and properties of three inducible bactericidal proteins from hemolymph of immunized pupae of Hyalophora cecropia Eur J Biochem 1980 106 7 16 7341234 10.1111/j.1432-1033.1980.tb05991.x

[B12] Christophides GK Zdobnov E Barillas-Mury C Birney E Blandin S Blass C Brey PT Collins FH Danielli A Dimopoulos G Immunity-related genes and gene families in Anopheles gambiae Science 2002 298 159 165 10.1126/science.1077136 12364793

[B13] Hoffmann JA Kafatos FC Janeway CA Ezekowitz RAB Phylogenetic perspectives in innate immunity Science 1999 284 1313 1318 10.1126/science.284.5418.1313 10334979

[B14] Chai LQ Tian YY Yang DT Wang JX Zhao XF Molecular cloning and characterization of a C-type lectin from the cotton bollworm, Helicoverpa armigera Dev Comp Immunol 2008 32 71 83 10.1016/j.dci.2007.04.006 17568670

[B15] Tian YY Liu Y Zhao XF Wang JX Characterization of a C-type lectin from the cotton bollworm, Helicoverpa armigera Dev Comp Immunol 2009 33 772 9 10.1016/j.dci.2009.01.002 19185587

[B16] Ribeiro C Brehélin M Insect haemocytes: what type of cell is that? J Insect Physiol 2006 52 417 29 10.1016/j.jinsphys.2006.01.005 16527302

[B17] Ling E Yu X Prophenoloxidase binds to the surface of hemocytes and is involved in hemocyte melanization in *Manduca sexta* Insect Biochem Mol Biol 2005 35 10.1016/j.ibmb.2005.08.007 16291091

[B18] Steiner H Hultmark D Engstrom A Bennich H Boman HG Sequence and specificity of two antibacterial proteins involved in insect immunity Nature 1981 292 246 8 10.1038/292246a0 7019715

[B19] Lee Y Yun E Jang W Kim I Lee J Park S Ryu K Seo S Kim C LI H Purification, cDNA cloning and expression of an insect defensin from the great wax moth, *Galleria mellonella* Insect Mol Biol 2004 13 65 72 10.1111/j.1365-2583.2004.00462.x 14728668

[B20] Mackintosh JA Gooley AA Karuso PH Beattie AJ Jardine DR Veal DA A gloverin-like antibacterial protein is synthesized in Helicoverpa armigera following bacterial challenge Developmental and Comparative Immunology 1998 22 387 399 10.1016/S0145-305X(98)00025-1 9699484

[B21] Fiolka MJ Ptaszynska AA Czarniawski W Antibacterial and antifungal lysozyme-type activity in Cameraria ohridella pupae Journal of Invertebrate Pathology 2005 90 1 9 10.1016/j.jip.2005.06.015 16169556

[B22] Hara S Yamakawa M Moricin, a novel type of antibacterial peptide isolated from the silkworm, *Bombyx mori* J Biol Chem 1995 270 29923 29927 10.1074/jbc.270.50.29923 8530391

[B23] Selisko B Garcia C Becerril B Gomez-Lagunas F Garay C Possani LD Cobatoxins 1 and 2 from Centruroides noxius Hoffmann constitute a subfamily of potassium-channel-blocking scorpion European Journal of Biochemistry 1998 254 468 479 10.1046/j.1432-1327.1998.2540468.x 9688256

[B24] Carlsson A Engström P Palva E H B Attacin, an antibacterial protein from *Hyalophora cecropia*, inhibits synthesis of outer membrane proteins in *Escherichia coli *by interfering with omp gene transcription Infect Immun 1991 59 3040 3045 1715318 10.1128/iai.59.9.3040-3045.1991 PMC258132

[B25] Schuhmann B Seitz V Vilcinskas A Podsiadlowski L Cloning and expression of gallerimycin, an antifungal peptide expressed in immune response of greater wax moth larvae, Galleria mellonella Archives of Insect Biochemistry and Physiology 2003 53 125 133 10.1002/arch.10091 12811766

[B26] Yoshida H Kinoshita K Ashida M Purification of a peptidoglycan recognition protein from hemolymph of the silkworm, Bombyx mori J Biol Chem 1996 271 13854 60 10.1074/jbc.271.23.13854 8662762

[B27] Lee WJ Lee JD Kravchenko VV Ulevitch RJ Brey PT Purification and molecular cloning of an inducible gram-negative bacteria-binding protein from the silkworm, Bombyx mori Proc Natl Acad Sci USA 1996 93 7888 93 10.1073/pnas.93.15.7888 8755572 PMC38844

[B28] Lemaitre B Reichart J-M Hoffmann J *Drosophila *host defense: differential induction of antimicrobial peptide genes after infection by various classes of microorganisms Proc Natl Acad Sci USA 1997 94 14614 14619 10.1073/pnas.94.26.14614 9405661 PMC25070

[B29] Li H Li WX Ding SW Induction and suppression of RNA silencing by an animal virus Science 2002 296 1319 21 10.1126/science.1070948 12016316

[B30] Hedges LM Johnson KN Induction of host defence responses by Drosophila C virus J Gen Virol 2008 89 1497 501 10.1099/vir.0.83684-0 18474566

[B31] Dostert C Jouanguy E Irving P Troxler L Galiana-Arnoux D Hetru C Hoffmann JA Imler JL The Jak-STAT signaling pathway is required but not sufficient for the antiviral response of drosophila Nat Immunol 2005 6 946 53 10.1038/ni1237 16086017

[B32] Zhao X-F Wang J-X Wang Y-C Purification and characterization of a cysteine proteinase from eggs of the cotton boll worm, *Helicoverpa armigera* Insect Biochem Mol Biol 1998 28 259 264 10.1016/S0965-1748(98)00015-0

[B33] Shao H-L Dong D-J Hu J-D Zhang X-C Zhang Y-B Fu Q Sun C Wang J-X Zhao X-F Reconstruction of AcMNPV with *Helicoverpa *hormone receptor 3 and its effect on the *Helicoverpa *larvae Biocontrol Sci Techn 2007 17 95 104 10.1080/09583150600937022

